# Correlation between the intestinal microflora and peripheral blood Th1/Th2 balance in hypothyroidism during the first half of pregnancy

**DOI:** 10.3389/fcimb.2023.1159238

**Published:** 2023-03-27

**Authors:** Bo Wu, Yajuan Xu, Yanjie Ban, Miao Zhang, Zongzong Sun, Yanjun Cai, Jingjing Li, Yingqi Hao, Qian Ouyang, Lin Hu, Xin Tian, Dong Liu

**Affiliations:** Department of Obstetrics and Gynecology, The Third Affiliated Hospital of Zhengzhou University, Zhengzhou, China

**Keywords:** hypothyroidism during pregnancy, Th1 cell, Th2 cell, cytokines, intestinal microflora

## Abstract

**Objective:**

This study aimed to investigate the relationship between intestinal microflora characteristics and the peripheral blood T helper cell (Th)1/Th2 balance in patients with hypothyroidism during the first half of pregnancy.

**Methods:**

The Th1/Th2 ratios in the peripheral blood of pregnant women in the hypothyroidism and control groups were determined using flow cytometry. The cytometric bead array assay was used to determine the serum levels of interleukin-2 (IL-2), IL-4, IL-6, IL-10, tumor necrosis factor (TNF)-α, and interferon (IFN)-γ. Moreover, 16S rRNA amplicon sequencing was used to determine the intestinal microbial composition in the two groups. Finally, the relationships between intestinal microflora, Th1/Th2 cells, cytokines, and clinical indicators were analyzed.

**Results:**

C-reactive protein levels were higher in the hypothyroidism group than in the control group. In contrast to the control group, the hypothyroidism group showed an increase in Th1 cells and the Th1/Th2 ratio, and a decrease in Th2 cells. The hypothyroidism group had higher serum IL-2, TNF-α, and IFN-γ levels, and lower IL-10 levels, than the control group. The richness of the intestinal microflora in the hypothyroidism group increased whereas the diversity decreased. The linear discriminant analysis effect size revealed that the hypothyroidism group had a higher abundance of *Prevotella* and *Faecalibacterium*, but a lower abundance of *Bacteroides*, compared to the control group. *Prevotella* was positively correlated with Th1 cells, the Th1/2 ratio, and TNF-α. *Bacteroides* was positively correlated with Th2 cells and IL-10, but negatively correlated with Th1 cells, the Th1/2 ratio, TNF-α, and IFN-γ. The thyroid peroxidase antibody level was directly proportional to TNF-α.

**Conclusion:**

A Th1/Th2 imbalance occurs in patients with hypothyroidism during the first half of pregnancy. Disorders of the intestinal microflora may lead to hypothyroidism during pregnancy by affecting the Th1/Th2 balance.

## 1 Introduction

Hypothyroidism during pregnancy is a systemic hypometabolic syndrome caused by thyroid hormone resistance or hypothyroxinemia, with an incidence of 0.2%–0.5%. However, its specific pathogenesis remains unclear (Świątkowska-Stodulska et al., 2022). During the first half of pregnancy (i.e., before 20 weeks of gestation), the fetal brain rapidly develops in conjunction with the development and migration of neural tissues. At this stage, the fetal thyroid gland is not yet fully active, and thyroid hormones that are essential for brain development are mainly derived from the mother (Huget-Penner and Feig, 2020; Geno and Nerenz, 2022; Lee and Pearce, 2022). Therefore, maternal hypothyroidism causes irreversible damage to fetal neurodevelopment and increases the risk of adverse pregnancy outcomes (Huget-Penner and Feig, 2020; Geno and Nerenz, 2022; Lee and Pearce, 2022; Świątkowska-Stodulska et al., 2022).

Autoimmune thyroid disease is the leading cause of hypothyroidism during pregnancy when iodine intake is sufficient (Chiovato et al., 2019). Hypothyroidism can significantly affect the function of the immune system (Adamczewski et al., 2020; Jaeger et al., 2021), and the infiltration of T lymphocytes can directly lead to the destruction of thyroid tissue (Li et al., 2019). Helper T (Th) cells secrete various cytokines; Th1 cells secrete proinflammatory cytokines such as interleukin (IL)-2, tumor necrosis factor (TNF)-α, and interferon (IFN)-γ, whereas Th2 cells antagonize the function of Th1 cells by secreting IL-4, IL-6, and IL-10. The Th1/Th2 balance is crucial for maintaining the normal immune function of the body; a Th1/Th2 imbalance will lead to immune dysfunction, thereby causing autoimmune diseases (Zhu and Zhu, 2020; Butcher and Zhu, 2021).

A growing body of evidence suggests that intestinal microflora disorders can lead to various diseases caused by autoimmune and inflammatory responses through an imbalance in T cell subsets, such as Th1 and Th2 cells (Brown et al., 2019; Kageyama et al., 2020). We previously demonstrated that the intestinal microflora is significantly altered in patients with hypothyroidism during the third trimester of pregnancy, and that changes in the microbiome may be involved in the development of hypothyroidism during pregnancy (Wang et al., 2020; Cai et al., 2021). However, to date, there are few reports on intestinal microflora characteristics and peripheral Th1/Th2 balance in patients with hypothyroidism in the first half of pregnancy.

The present study aimed to analyze the characteristics of the intestinal microflora in pregnant women with hypothyroidism in the first half of pregnancy, and its relationship with the Th1/Th2 balance and cytokines in the peripheral blood, to investigate the immunopathogenesis of hypothyroidism during pregnancy.

## 2 Materials and methods

### 2.1 Study patients

A total of 57 pregnant women with hypothyroidism in the first half of pregnancy (hypothyroidism group) and 58 healthy pregnant women during the same period (control group) were recruited from the Third Affiliated Hospital of Zhengzhou University between December 2021 and August 2022. The thyroid function levels of the hypothyroidism group were determined following the 2017 guidelines of the American Thyroid Association for the diagnosis and management of thyroid disease during pregnancy and the postpartum period (Alexander et al., 2017). The diagnostic criteria were established by the Clinical Laboratory of the Third Affiliated Hospital of Zhengzhou University [free T4 (FT4, thyroxine) < 12.3 pmol/L, serum thyroid stimulating hormone (TSH) > 4.2 mIU/L]. All pregnant women in the hypothyroidism group were first diagnosed with hypothyroidism when they came to our hospital. Patients without other obstetric or thyroid complications were included in the control group. All patients were in the first half of their pregnancy (gestational age < 20 weeks). The exclusion criteria were as follows: age < 18 years; combined immune system disease or endocrine disease; severe stress, anxiety, and depression; artificial conception and multiple pregnancies; history of gastrointestinal surgery; received antibiotic, probiotic, or immunosuppressant treatment in the previous 2 months; received antithyroid drugs or thyroid hormone replacement; and other pregnancy complications. The pregnant women in both groups were residents of Zhengzhou and had similar diets. All participants provided written informed consent before participation. Ethical approval was granted by the Third Affiliated Hospital of Zhengzhou University, China.

### 2.2 Collection of blood and stool samples

Each patient fasted for 8–12 h overnight before the collection of blood samples. Two 5-mL samples of blood were collected from the median cubital vein. One 5-mL sample was stored at 4°C for flow cytometry analysis, which was performed within 6 h after collection. The other blood sample was centrifuged at 1600 g for 10 min for the cytometric bead array assay, and the serum (0.5 mL) was collected for analyses, which were completed within 4 h of collection.

All patients were informed of the following precautions prior to stool collection: high-fat foods should not be consumed for 3 days; lactic acid products such as yogurt and probiotic-related microbial products should not be consumed for 5 days; and oral laxatives should not be taken for 1 week. Samples were collected carefully from the middle of the stool, which had not been in contact with the bedpan, avoiding mixing with urine or contact between the sterile spoon and other parts of the sample. The samples were placed in a 2.0-mL sterile tube after collection and stored in a −80°C refrigerator within 2 h.

### 2.3 Flow cytometry analysis

Th1 and Th2 cells were quantified using intracellular cytokine staining. Heparin sodium-anticoagulated peripheral blood (200 μL) was added into a 96-well plate, and phorbol myristate acetate (50 ng/mL), ionomycin (1μg/mL), and brefeldin A (10 μg/mL) were added. Blood samples were cultured for 4 h at 37°C under 5% CO_2_. Surface-labeled antibodies (i.e., APC-CY7-CD3 (clone SP34-2) and FITC-CD4 (clone RPA-T4)) were added. After 20 min of incubation in the dark, the erythrocyte lysis solution was added, and the whole mixture was incubated for 15 min. The supernatant was discarded after centrifugation at 500 g for 5 min, washed, and centrifuged again. A membrane-breaking fixative solution was then added and incubated for 50 min in the dark. Antibodies against PE-IFN-γ (clone 4S. B3) and PE-CY7-IL-4 (clone 8D4-8) were added for intracellular staining, and the cells were incubated in the dark for 50 min. Phosphate-buffered saline (PBS, 2 mL) was added, and the cells were then centrifuged for 5 min at 500 g, resuspended in 500 μL of PBS, and then subjected to a flow cytometric analysis. Flow cytometry data were analyzed using FlowJo software (Tree Star, Ashland, OR, USA). Fluorescent Minus One samples were used as a gating strategy control. All antibodies were obtained from BD Biosciences (Franklin Lakes, NJ, USA).

### 2.4 Cytokine detection

The levels of IL-2, IL-4, IL-6, IL-10, TNF-α, and IFN-γ were measured using a human Th1/Th2 cytokine kit (Nord, Jiangxi, China) following the manufacturer’s protocol. Briefly, capture antibody-coupled beads were combined with 25 μL of serum, and the mixture was then incubated with 25 μL of fluorescent-labeled detection antibodies for 2.5 h at 20−25°C in the dark. PBS was used to wash and resuspend the beads, and the samples were analyzed using flow cytometry.

### 2.5 16S rRNA gene sequencing

Approximately 0.2 g of the stool specimens were collected, and a KF kit B (Magen Biotechnology, Guangzhou, China) was used to extract the total DNA of the bacterial populations. The Qubit dsDNA BR Assay Kit (Invitrogen, USA, cat: Q32850) was used to measure the concentration of DNA. The polymerase chain reaction (PCR) was used to amplify the 16S rRNA V3–V4 region. The primers used in the PCR were 338F (5’-ACTCCTACGGGAGGCAGCAG-3’) and 806R (5’-GGACTACHVGGGTWTCTAAT-3’). After purification, magnetic beads were used to verify the quality of the samples. The Miseq Reagent Kit v3 (Illumina, USA) was used for library construction. The Illumina MiSeq PE300 platform (BGI, Shenzhen, China) (Fadrosh et al., 2014) was used for sequencing to generate 300-bp paired-end reads. After obtaining the Illumina raw data in fastq format, each fastq file was processed using FastQC software (v0.11.2). The final Q20 and Q30 of all samples were above 90% and 85%, respectively, after data filtering for quality control. Fast Length Adjustment of SHort reads (v1.2.11) (Magoč and Salzberg, 2011) was used to splice the sequences. The pairs of reads sequenced from the double ends were assembled into a sequence using the overlap relationship, and the Tags of the highly variable region were obtained. Operational taxonomic unit (OTU) generation and clustering were performed with a cut-off of 97% similarity using USEARCH (v7.0.1090) (Edgar, 2013). The representative sequence for each OTU was annotated using the RDP database (release 11, update 5 dated September 30, 2016) with a confidence threshold of 0.6. Based on the results of species annotation and classification, alpha diversity, beta diversity, partial least squares-discriminant analysis (PLS-DA), and linear discriminant analysis (LDA) effect size (LEfSe) analysis was determined to analyze the differences between groups. Kyoto Encyclopedia of Genes and Genomes (KEGG) pathways were then predicted by PICRUST software (Wilkinson et al., 2018). Intestinal microflora with significant differences between groups were further analyzed by a receiver operating characteristic (ROC) curve analysis.

### 2.6 Data collection

We collected data on maternal age, body mass index (BMI) at enrollment, gestational age, gravidity, and parity on the day of enrollment. Clinical data, such as the levels of TSH, FT4, C-reactive protein (CRP), and thyroid peroxidase antibody (TPOAb) levels, were determined when collecting fecal and blood samples.

### 2.7 Statistical analyses

Categorical variables are described as frequencies, normally distributed data are described as means with standard deviations, while data that were not normally distributed are reported as medians and quartiles. Student’s *t*-test and the Mann–Whitney U test were used to compare differences between the two groups. Alpha diversity, beta diversity, and KEGG modules were tested using the Wilcoxon rank sum test. Based on the PLS-DA results, LEfSe analysis was performed to assess the differences in the microflora abundance between groups. Statistical analyses were conducted using Prism software (GraphPad, La Jolla, CA, USA). The correlation analysis was performed using Spearman’s correlation coefficient and redundancy analysis (RDA), and statistical significance was set as *p* < 0.05.

## 3 Results

### 3.1 General clinical data

Differences in age, BMI at enrollment, gestational weeks, gravidity, and parity were not significant between the hypothyroidism and control groups (*p* > 0.05). However, compared to the control group, higher serum CRP (*p* = 0.003) and TPOAb (*p* = 0.004) levels were observed in the hypothyroidism group (**Table 1**).

**Table 1:** General clinical data of the hypothyroidism and control groups.

	Hypothyroidism group (n = 57)	Control group (n = 58)	P-value
Age* (years)	29.30 ± 3.21	29.67 ± 2.81	0.507
BMI at enrollment* (kg/m^2^)	22.70 ± 2.21	22.73 ± 2.50	0.943
Gestational weeks**^#^** (weeks)	13.57 (10.22, 17.29)	13.71 (11.11, 17.14)	0.792
Gravidity**^#^** (n)	2.00 (1.00, 3.00)	2.00 (1.00, 3.00)	0.247
Parity* (n)	0.63 ± 0.67	0.72 ± 0.81	0.507
FT4**^#^** (pmol/L)	11.00 (10.34, 11.65)	16.65 (13.85, 18.13)	< 0.001
TSH**^#^** (mIU/L)	5.10 (4.51, 5.73)	1.78 (1.34, 2.45)	< 0.001
TPOAb**^#^** (U/mL)	35.00 (9.42, 49.45)	13.55 (10.07, 23.20)	0.004
CRP**^#^** (mg/L)	2.42 (1.55, 4.58)	1.86 (0.98, 3.27)	0.003

^*^ Data are presented as means ± standard deviation.

^#^Data are presented as medians (P25, P75).

P < 0.05 was considered statistically significant.

BMI, body mass index; FT4, free T4; TSH, thyroid-stimulating hormone; TPOAb, thyroid peroxidase antibody; CRP, c-reactive protein.

### 3.2 Th1 and Th2 levels

**Figure 1:**
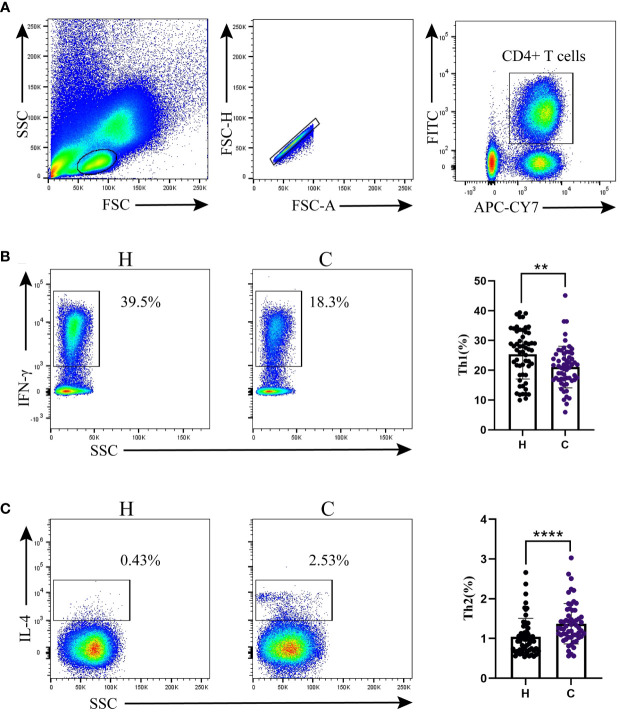
Th1/Th2 detection by flow cytometry. **(A)** Flow cytometry gating strategy. Percentages of **(B)** Th1 cells and **(C)** Th2 cells. Differences between the two groups were determined using the Mann–Whitney U test. H: Hypothyroidism group. C: Control group. ^**^ *p* < 0.01; ^****^ *p* < 0.0001.

The gating strategy is shown in **Figure 1A**. CD4+ T cells were gated to lymphocytes expressing CD3 and CD4. We quantified Th1 and Th2 cells by measuring the expression levels of IFN-γ and IL-4. Compared to the control group, the hypothyroidism group had a higher percentage of Th1 cells (*p* < 0.01, **Figure 1B**), a higher Th1/Th2 ratio (*p* < 0.001, **Table 2**), and a lower percentage of Th2 cells (*p* < 0.001, **Figure 1C**).

**Table 2:** Comparison of Th1/Th2 cells between groups.

	Hypothyroidism group (n = 57)	Control group (n = 58)	P-value
Th1 (%)	26.80 (18.45, 32.65)	21.15 (16.80, 25.45)	0.002^**^
Th2 (%)	0.93 (0.70, 1.21)	1.27 (1.05, 1.61)	< 0.001^****^
Th1/Th2	26.53 (16.11, 42.68)	15.74 (11.23, 21.26)	< 0.001^****^

Data are presented as means ± standard deviation. ^**^ p < 0.01, ^****^ p < 0.0001.

### 3.3 Serum cytokine concentrations

The serum levels of IL-2, TNF-α, and IFN-γ in the hypothyroidism group were significantly increased (*p* < 0.01, *p* < 0.001, *p* < 0.001, respectively), whereas the level of IL-10 was dramatically reduced (*p* < 0.001), compared to those in the control group. Both groups had similar serum levels of IL-4 and IL-6 (*p* > 0.05, **Table 3**).

**Table 3:** Comparison of serum cytokine concentrations between groups.

	Hypothyroidism group (n = 57)	Control group (n = 58)	P- value
IL-2 (pg/mL)	1.64 (1.19, 3.04)	1.21 (0.58, 1.66)	0.003^**^
IL-4 (pg/mL)	1.25 (0.52, 2.05)	1.44 (0.89, 2.64)	0.179
IL-6 (pg/mL)	2.27 (1.34, 3.27)	2.29 (1.59, 3.91)	0.387
IL-10 (pg/mL)	0.99 (0.61, 1.47)	2.27 (1.53, 3.78)	< 0.001^****^
TNF-α (pg/mL)	2.00 (1.21, 3.91)	1.20 (0.87, 1.51)	< 0.001^****^
IFN-γ (pg/mL)	2.16 (1.46, 2.99)	1.44 (1.05, 1.81)	< 0.001^***^

Data are expressed as medians (P25, P75). ^**^ p < 0.01, ^***^ p < 0.001, ^****^ p < 0.0001.

IL, interleukin; TNF-α, tumor necrosis factor alpha; IFN-γ, interferon-gamma.

### 3.4 Characteristics of the intestinal microflora

**Figure 2:**
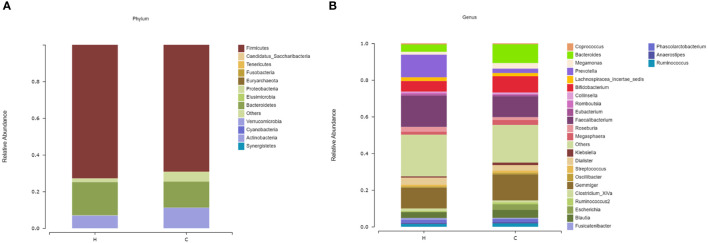
The microbial compositions of the hypothyroidism and control groups were compared at different levels. **(A)** Phylum level. **(B)** Genus level. The species whose abundances were less than 0.5% in the samples were classified into “others”. H, Hypothyroidism group; C, Control group.

**Figure 3:**
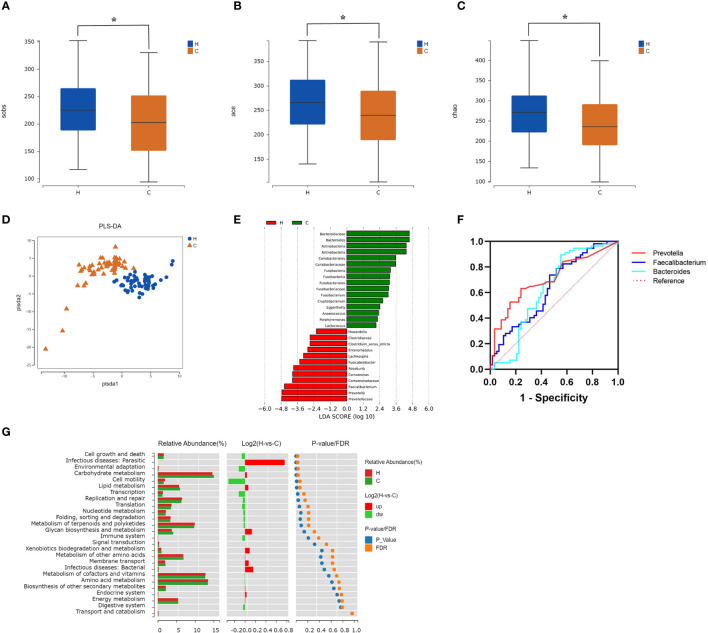
Comparison of the intestinal microflora characteristics between the hypothyroidism and control groups. **(A–C)** The alpha diversity (Sobs index, Ace index, Chao index) of the two groups revealed statistically significant differences ( ^*^ *p* < 0.05). **(D)** The partial least squares-discriminant analysis model score plot. Each dot corresponds to one sample. **(E)** LDA chart. The LDA effect size analysis was used for scoring. Enriched taxa in the control group are represented by positive LDA scores (green), and enriched taxa in the hypothyroidism group are represented by negative LDA scores (red). A higher LDA score indicates a greater influence on differences between groups. Taxa with LDA scores > 2 are displayed (*p* < 0.05). **(F)** Receiver operating characteristic curves of *Prevotella*, *Faecalibacterium*, and *Bacteroides* (*p* < 0.05). **(G)** Kyoto Encyclopedia of Genes and Genomes pathway enrichment results. The bar chart on the left displays the relative abundance of pathways in the two groups. The log2 value in the middle represents the mean ratio of relative abundance of the same pathway in the two groups. The right figure shows the *p*-value and FDR values by the Wilcoxon rank-sum test. If the *p*-value and FDR values were < 0.05, the pathway was significantly different between the two groups. H, Hypothyroidism group; C, Control group.

*Firmicutes* and *Bacteroidetes* were dominant in both groups at the phylum level (**Figure 2A**); however, there was no significant difference in the *Firmicutes/Bacteroidetes* (F/B) ratio between the two groups (*p* = 0.839, **Supplementary Table 1**). At the genus level, the most abundant microflora were *Faecalibacterium*, *Gemmiger*, *Bifidobacterium*, and *Bacteroides* in the two groups (**Figure 2B**). Compared to the control group, the Sobs, Ace, and Chao indices in the hypothyroidism group were significantly increased (*p* = 0.015, *p* = 0.011, and *p* = 0.016, respectively; **Figures 3A–C**; **Supplementary Table 2**). There were no statistically significant differences in the Shannon (Shannon–Weaver) and Simpson indices between the two groups (*p* = 0.829 and *p* = 0.205, respectively; **Supplementary Table 2**). The PLS-DA revealed that the samples could be significantly separated into two areas (**Figure 3D**). The LEfSe analysis revealed that the abundance of *Prevotella* and *Faecalibacterium* was strikingly increased in the hypothyroidism group, whereas that of *Bacteroides* was significantly increased in the control group. The LDA score revealed that *Prevotella* and *Bacteroides* were the most important characteristic genera among the top 16 significantly different genera between the two groups (*p* < 0.05; **Figure 3E**; **Supplementary Table 3**). In the ROC curve analysis, when the levels of *Prevotella*, *Faecalibacterium*, and *Bacteroides* were used to predict hypothyroidism during pregnancy, the areas under the curve were 0.71, 0.63, and 0.62, respectively (*p* < 0.05; **Figure 3F**; **Supplementary Table 4**). The function of the intestinal microflora was predicted using the KEGG database. Significant differences in the four functional pathways at the L2 level, including cell growth and death, infectious diseases, parasitic, environmental adaptation, and carbohydrate metabolism, were revealed between the two groups (*p* < 0.05, FDR < 0.05; **Figure 3G**; **Supplementary Table 5**).

### 3.5 Correlations between the intestinal microflora with Th1/Th2 cells, cytokines, and clinical indicators

**Figure 4:**
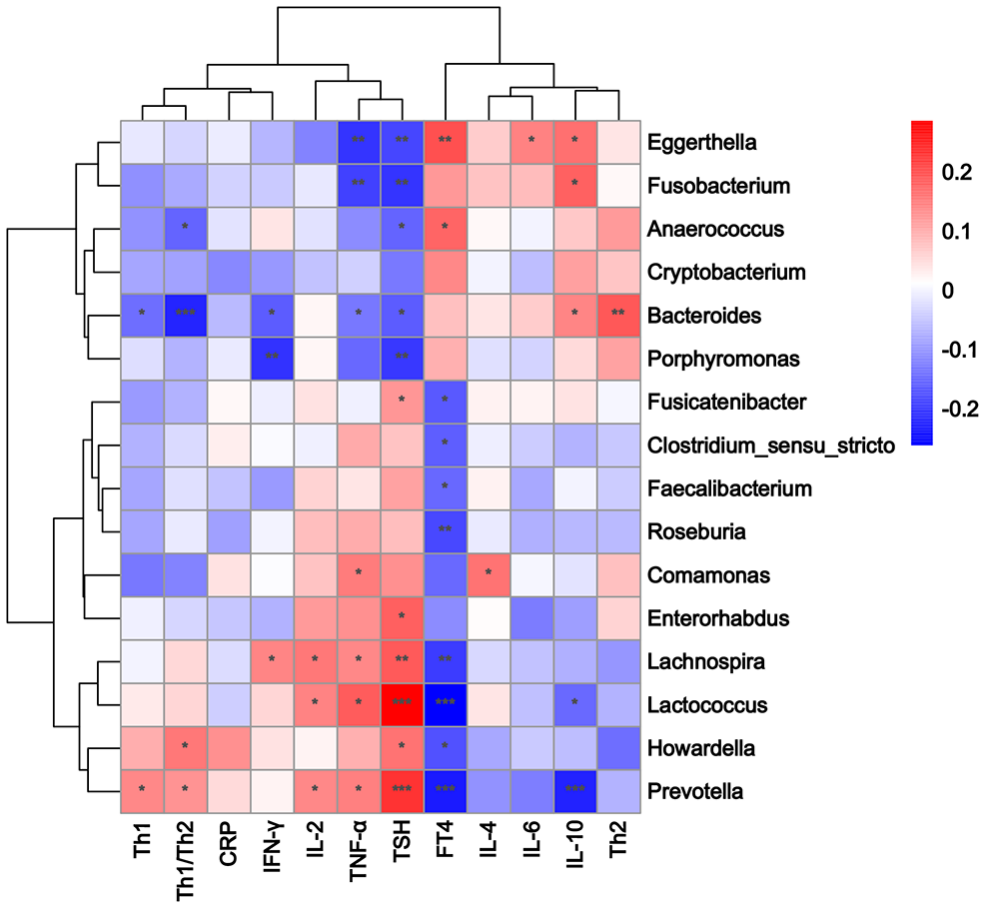
Heatmap of Spearman rank correlation coefficients between 16 different genera, Th1/Th2 cells, cytokines, and clinical indicators. Color intensity indicates the magnitude of correlation. Red depicts positive correlations, and blue depicts negative correlations. ^*^ *p* < 0.05, ^**^ *p* < 0.01, ^***^ *p* < 0.001.

**Figure 5:**
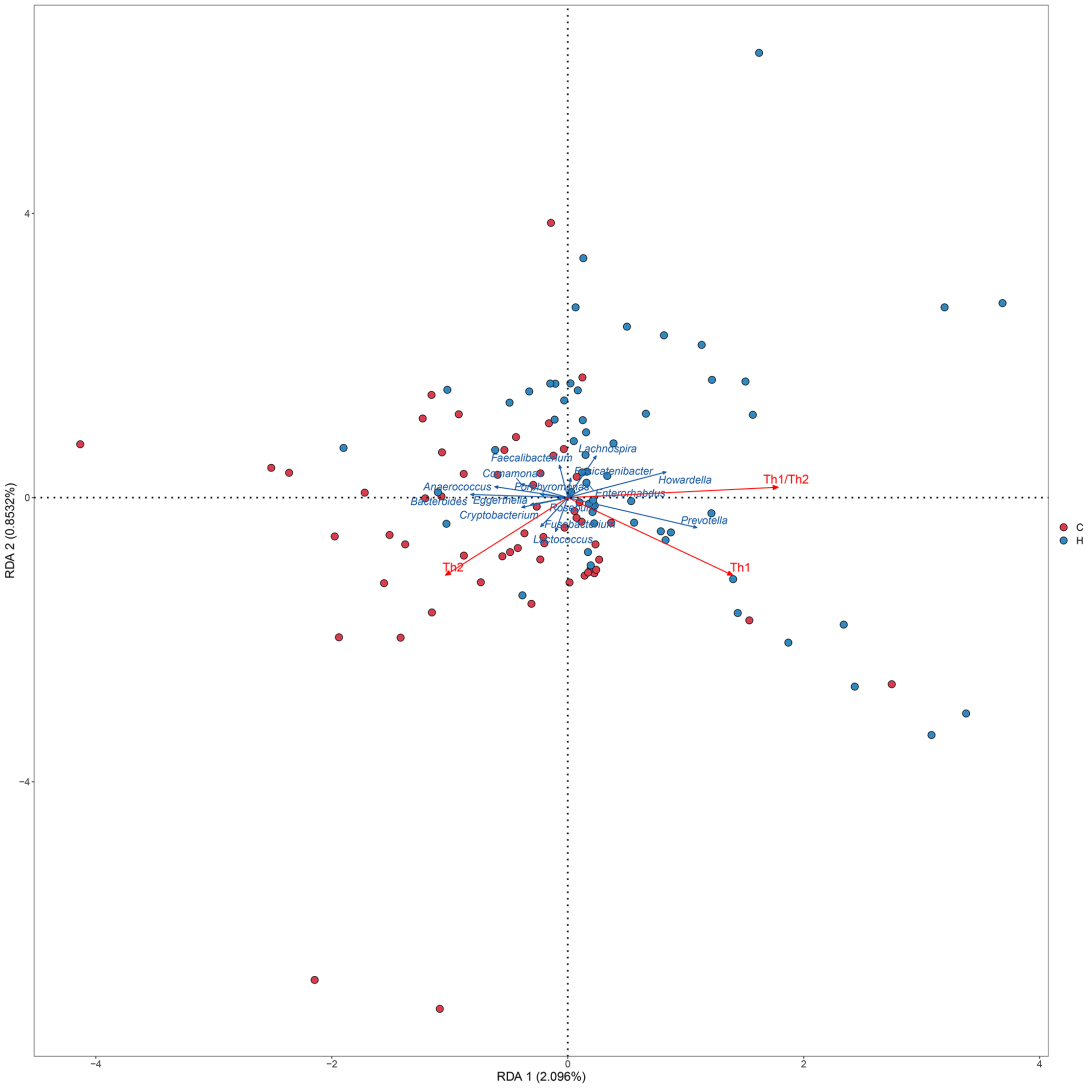
RDA between Th levels and microbiome. The dots indicate the samples, red indicates each sample in the hypothyroidism group, and blue indicates each sample in the control group. The red arrows indicate the Th cells and Th1/Th2 ratio, and the blue arrows indicate the different genera. The angle between the red arrow and the blue arrow represents the correlation between the Th cell, Th1/Th2 ratio and the different genera (Sharp angle, positive correlation; obtuse angle, negative correlation; right angle, no correlation). H, Hypothyroidism group; C, Control group.

Correlations among the 16 significantly different genera, Th1/Th2 cells, cytokines, and clinical indicators were analyzed using Spearman’s correlation analysis. A standard *p*-value < 0.05, *q* value < 0.1 was used to determine statistical significance. The correlation heatmap revealed that the abundance of *Prevotella* was positively correlated with Th1 cells (*p* = 0.030, *q* = 0.062), the Th1/Th2 ratio (*p* = 0.050, *q* = 0.077), and the serum TNF-α level (*p* = 0.022, *q* = 0.062). The abundance of *Bacteroides* was positively correlated with Th2 cells (*p* = 0.003, *q* = 0.015) and serum IL-10 levels (*p* = 0.024, *q* = 0.051), and negatively correlated with Th1 cells (*p* = 0.026, *q* = 0.051), the Th1/Th2 ratio (*p* < 0.001, *q* = 0.006), serum TNF-α (*p* = 0.037, *q* = 0.063), and the IFN-γ level (*p* = 0.011, *q* = 0.033; **Figure 4**; **Supplementary Table 6**). The TPOAb level was directly proportional to the serum TNF-α level (*p* = 0.030, *q* = 0.120; **Supplementary Table 7**). The RDA also showed that *Prevotella* was positively correlated with Th1 cells and the Th1/Th2 ratio, while *Bacteroides* was positively correlated with Th2 cells, and negatively correlated with Th1 cells and the Th1/Th2 ratio (**Figure 5**).

## 4 Discussion

A growing body of evidence suggests a link between thyroid homeostasis and intestinal microflora. Dysregulation of intestinal microflora affects normal intestinal function, leads to immune and metabolic disorders, and plays a crucial role in the occurrence and development of thyroid diseases (Ejtahed et al., 2020; Knezevic et al., 2020; Virili et al., 2021). The present study investigated the correlation between intestinal microflora and hypothyroidism during pregnancy from the perspective of immunity.

CRP is a sensitive marker of inflammation and tissue damage, and a high CRP level indicates an elevated proinflammatory response. In the present study, the serum CRP level was significantly increased in the hypothyroidism group, which is consistent with our previous findings (Cai et al., 2021). Therefore, the inflammatory response may contribute to the development of hypothyroidism during pregnancy.

In the first half of pregnancy, the balance of Th1/Th2 gradually shifts toward Th2 under the action of maternal hormones, which is called the “Th2 phenomenon” (Challis et al., 2009). In the current study, the number of Th1 cells and the Th1/Th2 ratio in pregnant women with hypothyroidism were significantly increased, whereas the number of Th2 cells was decreased. Our results are consistent with those of the study by Guo et al. (2018) reporting that patients with a higher proportion of Th1 cells are more inclined to experience rapid destruction of thyroid follicular cells, resulting in hypothyroidism. A predominance of the Th1 immune response accelerates apoptosis of thyroid cells mediated by Fas and TRAIL, which causes an immune imbalance and enhanced inflammatory response, ultimately leading to thyroid tissue injury (Stassi and De Maria, 2002; Fountoulakis et al., 2008). Accordingly, we believe that a shift in the Th1/Th2 balance toward Th1 cells may affect thyroid function and promote hypothyroidism during pregnancy by inducing an autoimmune response or apoptosis of thyroid cells.

In the present study, serum IL-2, TNF-α, and IFN-γ levels were significantly increased in the hypothyroidism group compared to those in the control group. Similarly, Ragusa et al. have also reported that thyroid diseases are associated with these cytokines. The main possible mechanisms underlying this phenomenon are as follows: (a) IL-2 and IFN-γ may participate in the thyroid autoimmune process and affect thyroid function (Krieg et al., 2018) by blocking the PD-1/PD-L1 pathway; (b) IFN-γ and TNF-α produced by Th1 cells induce the release of CXCL10 from thyroid cells. CXCL10 then binds to the CXCR3 receptor on Th1 cells (Ragusa et al., 2019), shifting the Th1/Th2 balance toward Th1 dominance; and (c) IFN-γ and TNF-α can also stimulate thyroid cells to synthesize and release IFN-γ-inducible chemokines into the circulation, which elicits thyroid destruction and hypothyroidism through pyroptosis (Ragusa et al., 2019; Weetman, 2021).

Dysregulation of the intestinal microflora contributes to the occurrence and development of thyroid diseases (Ejtahed et al., 2020; Knezevic et al., 2020; Virili et al., 2021). Our results showed that there was no significant difference in the F/B ratio between the hypothyroidism and control groups. However, Su et al. (2020) found that the F/B ratio increased in patients with primary hypothyroidism. The reason for the discrepancy between our studies and theirs is unclear. This may be owing to the short duration of disease in our patients. In addition, pregnancy is a special period for women, during which endocrine and physiological fluctuations occur, which may have an impact on the intestinal microflora. In the present study, significant differences in alpha diversity were identified between the two groups *via* 16S rRNA gene amplicon sequencing. Moreover, higher Sobs, Ace, and Chao indices were observed in the hypothyroidism group, indicating higher intestinal community richness and suggesting that the richness of microflora is increased in pregnant women with hypothyroidism; this is consistent with a previous study (Su et al., 2020). A higher Simpson index (the higher the index, the lower the diversity) and lower Shannon index were observed in the hypothyroidism group, indicating that bacterial community diversity tended to decline, which is consistent with the notion that a higher diversity of the intestinal microflora is associated with improved overall health (Le Chatelier et al., 2013). We also revealed that the hypothyroidism group had a higher abundance of *Prevotella* and *Faecalibacterium*, and a lower abundance of *Bacteroides*, which is consistent with a previous study reporting that the abundance of *Prevotella* is inversely correlated with that of *Bacteroides* in the intestinal microflora, and the presence of one bacterium results in the exclusion of the other (Tett et al., 2021). In addition, the KEGG pathway analysis showed that the *p* and FDR values were the lowest in the hypothyroidism group, and that the targets were mostly enriched in the cell growth and death pathways. *Bacteroides* have been shown to facilitate peroxisome proliferator activated receptor-γ-mediated alterations in nuclear factor kappa B (NF-κB) activity (Li et al., 2021), and the dysregulation of NF-κB signaling leads to abnormal cell proliferation and resistance to programmed cell death signals (Beg and Baltimore, 1996). Lipopolysaccharides on the outer membrane of *Prevotella* can regulate the p38 MAPK, JAK-STAT, and NF-κB signaling pathways (Sharma et al., 2022), and thus participate in cell growth, apoptosis, and death (Beg and Baltimore, 1996; Yosimichi et al., 2001; Lu et al., 2008). This suggests that *Bacteroides* and *Prevotella* may influence the occurrence of hypothyroidism during pregnancy through the cell growth and death pathways.

In the present study, our correlation analysis revealed that the abundance of *Prevotella* was positively associated with Th1 cells, the Th1/Th2 ratio, and the TNF-α level. Lipopolysaccharides of *Prevotella*, a potent inducer of the inflammatory response, can induce the increase of several pro-inflammatory cytokines, such as TNF-α and IL-6 (Sharma et al., 2022). *Prevotella* can induce Th1 polarization and inhibit Th2 polarization through dendritic cells, thus causing the Th1/Th2 balance to shift to the left (van Teijlingen et al., 2020). An immune imbalance and enhanced inflammatory response can destroy thyroid follicular cells, eventually leading to hypothyroidism. Moreover, we found that the abundance of *Bacteroides* was positively associated with Th2 cells and IL-10 levels, and negatively associated with Th1 cells, the Th1/Th2 ratio, and TNF-α and IFN-γ levels. An animal experiment has demonstrated that *Bacteroides* can increase the proportion of Th1 cells and decrease the proportion of Th2 cells by regulating the expression of related transcription factors, resulting in a left shift of the Th1/Th2 balance (Li et al., 2021). *Bacteroides* can also attenuate the expression of pro-inflammatory cytokines such as IL-1β and TNF-α, providing anti-inflammatory effects (Kelly et al., 2004). In addition, *Bacteroides* can boost antigen-specific Th1 cells to secrete IL-10 and limit the release of IFN-γ, thereby weakening the pathogenic potential of Th1 cells in the inflammatory response (Sun et al., 2018), which is related to the occurrence of hypothyroidism during pregnancy.

Our correlation analysis revealed that the TPOAb level was directly proportional to that of TNF-α. Karanikas et al. (2005) have shown that a high titer of TPOAb may significantly contribute to increased TNF-α production, which is associated with high activity of Hashimoto thyroiditis. Therefore, we suggest that a disturbance of intestinal microflora components, such as *Prevotella* and *Bacteroides*, can affect the Th1/Th2 balance and related cytokines, and an increase in the levels of cytokines, such as TNF-α and IFN-γ, can cause thyroid injury *via* various pathways. TPOAb may aggravate the thyroid injury process by enhancing the production of TNF-α, thereby affecting thyroid function and leading to hypothyroidism.

Our study has some limitations. The results may be biased without a sufficient sample size to reveal the relationship between intestinal microflora and immune indicators. Furthermore, different regions or countries have different dietary habits, which makes our results regionally specific. Finally, the mechanisms by which hypothyroidism during pregnancy leads to alterations in the intestinal microflora and the Th1/Th2 balance have not been addressed. Therefore, it is necessary to conduct further mechanistic studies to better understand the pathogenic implications of the intestinal microflora and the Th1/Th2 balance in pregnant women with hypothyroidism.

In summary, we investigated the correlation between hypothyroidism during pregnancy and intestinal microflora at the immune level by determining changes in the peripheral blood Th1/Th2 balance and intestinal microflora of the two groups. The results revealed that *Prevotella* and *Bacteroides* may participate in the occurrence and development of hypothyroidism during pregnancy by affecting the Th1/Th2 balance and related cytokines. This study provides new insights into the mechanisms underlying hypothyroidism during pregnancy from an immunological perspective.

## Data availability statement

The data presented in the study are deposited in the NCBI SRA repository (accession number PRJNA934339) and Figshare repository (10.6084/m9.figshare.21561891).

## Ethics statement

The studies involving human participants were reviewed and approved by the Ethics Committee of the Third Affiliated Hospital of Zhengzhou University. The patients/participants provided their written informed consent to participate in this study.

## Author contributions

BW and LH performed the experiments. BW and YC analyzed the data. BW, QO, YH, ZS, LH, XT, and DL collected samples for the experiments. YX and BW wrote the manuscript. MZ, JL, and YB revised the manuscript. All authors contributed to the article and approved the submitted version.

## Supplementary material

Supplementary Table 1 Relative abundance of sample species.

Supplementary Table 2 Alpha diversity of the sample.

Supplementary Table 3 The LDA score.

Supplementary Table 4 The areas under the curve.

Supplementary Table 5 The relative abundance and p value of functional pathways.

Supplementary Table 6 Correlations among the 16 different genera, Th1 cell, Th2 cell, cytokines, and clinical indicators.

Supplementary Table 7 Correlations among TPOAb, Th1 cell, Th2 cell, cytokines, and clinical indicators.
